# Sound Analysis to Predict the Growth of Turkeys

**DOI:** 10.3390/ani10050866

**Published:** 2020-05-17

**Authors:** El-Sayed M. Abdel-Kafy, Samya E. Ibraheim, Alberto Finzi, Sabbah F. Youssef, Fatma M. Behiry, Giorgio Provolo

**Affiliations:** 1 Animal Production Research Institute, Agricultural Research Center, Dokki, Giza 12651, Egypt; isamya80@gmail.com (S.E.I.); sabbah.farouk@yahoo.com (S.F.Y.); fatma.behiry@yahoo.com (F.M.B.); 2 Department of Agricultural and Environmental Sciences, Università degli Studi di Milano, 20133 Milano, Italy; alberto.finzi@unimi.it

**Keywords:** turkey production, peak frequency, vocalization, weight prediction, animal welfare

## Abstract

**Simple Summary:**

The protocols for manual weighing of turkeys are not practical on turkey farms because of the large body sizes, heavy weights and flighty nature of turkeys. The sounds turkeys make may be a proxy for bird weights, but the relationship between turkey sounds and bird weights has not been studied. The aim of this study was to correlate the sound of turkeys with their age and weight and examine the possibility of using sound to predict bird weights. The study consisted of four trials in Egypt. Sounds of birds and their weights were recorded for 11 days during the growth period in each trial. A total of 2200 sounds were used to manually analyze and label each sound using the peak frequency. There was a highly significant negative correlation between the peak frequency of vocalizations and the weight and age of the turkeys, showing that the peak frequency of vocalizations can be used for predicting the weight of turkeys.

**Abstract:**

Protocols for manual weighing of turkeys are not practical on turkey farms because of the large body sizes, heavy weights and flighty nature of turkeys. The sounds turkeys make may be a proxy for bird weights, but the relationship between turkey sounds and bird weights has not been studied. The aim of this study was to correlate peak frequency (PF) of vocalization with the age and weight of the bird and examine the possibility using PF to predict the weight of turkeys. The study consisted of four trials in Egypt. Sounds of birds and their weights were recorded for 11 days during the growth period in each trial. A total 2200 sounds were manually analyzed and labelled by extracting individual and general sounds on the basis of the amplitude and frequency of the sound signal. The PF of vocalizations in each trial, as well as in pooled trails, were evaluated to determine the relationship between PF and the age and weight of the turkey. PF exhibited a highly significant negative correlation with the weight and age of the turkeys showing that PF of vocalizations can be used for predicting the weight of turkeys. Further studies are necessary to refine the procedure.

## 1. Introduction

With rapid population expansion, improved quality of life and urban development in most parts of the world, agricultural development is playing an increasingly important role in the global economy [1]. Livestock/crop production is becoming increasingly industrialized worldwide, shifting from extensive, small-scale production systems towards more intensive, large-scale, specialized and commercially oriented enterprises [2]. According to the Food and Agriculture Organization (FAO) global turkey meat production increased by 13.5% between 2011 and 2018 as output rose from 5.2 to 5.9 million tons [3]. Due to selective breeding, turkeys now grow faster, reach slaughter weight at a younger age, and yield more edible meat than previously [4]. Also, application of welfare assessments in turkey production is commonplace and these measures have important impacts on a producer’s economic revenue [5]. Turkey weights are important as they relate to the health and welfare of birds [6]. Animal weight also is an economically important feature in livestock breeding. Weight is used as an indication about the general state of welfare of an animal, as well as about the biological parameters that influence the physical growth and health of animals [7]. Weight also is used to identify the optimum slaughtering age and to investigate the effects of selection on growth curve parameters [8].

The protocols for weighing turkeys require corralling and physically handling the birds. However, these methods are not practical on commercial turkey farms because the large body sizes, heavy weights and activity and flighty (i.e., erratic) nature of turkeys make their handling difficult and dangerous for the birds and handlers. Besides, manually measuring the weight of a turkey requires labor and deprives the farmer of useful time. Therefore, non-contact methods to record the weight of turkeys during their growth should be developed both to support producers and to promote animal welfare. For this purpose, the use of automatic animal monitoring or precision livestock farming (PLF) tools may be useful for collecting information about physiological responses [9], growth [10], social interactions [11], health and welfare status [12] and vocalizations [13] of the turkeys. Development and validation of easy-to-apply methodologies such as PLF tools for weighing and welfare assessment of turkeys is a critical step for reducing economic losses [14].

One possible PLF tool is audio data; analysis of bird sounds has been widely used in a large number of bird studies [15]. This technique may resolve many of the challenges associated with the weighing and assessment of welfare in turkey flocks. For example, Liu et al. [16] analyzed turkey sounds and showed that turkey vocal sounds could be successfully used as an early warning tool for heat stress detection. Fontana et al. [17] proposed a method to measure the growth of broiler chickens by analyzing the sounds they made. Their study showed a highly significant correlation (*p* ≤ 0.001) between the peak frequency (PF) of vocalization and the age and weight of broilers; they then used the relationship define a model for PF of vocalization and bird weight. Fontana et al. [18] validated the model of Fontana et al. [17] and developed it as an automatic tool for detecting the growth of chickens based on the PF of their vocalizations during the production cycle. Unfortunately, there is lack of an animal-based protocol for on-farm welfare assessment and weighing of commercially produced turkeys [19]. In fact, the relationship between turkey sounds and their weight has not yet been studied. Thus, the objective of this study was to analyze turkey vocalizations under normal commercial production conditions and correlate the analyzed sounds with the age and weight of the turkeys. Moreover, the possibility of using one or more specific parameters (such as PF) of the vocalizations as a predictor of the weight of the turkeys in their growing period (four months) was assessed on four different flocks.

## 2. Materials and Methods

### 2.1. Study Location and Ethics

The study monitored four groups of turkeys (i.e., four trials) and was conducted from December 2018 to February 2020. The trials were carried out in the research farm belonging to the Animal Production Research Institute (APRI), Agricultural Research Center (ARC), located in the north of Egypt. The Institute’s ethical rules for animal research were followed and the study plan was approved by the Institute’s Research Committee on 18 December 2017 (code no. 020203429).

### 2.2. Bird Management

Hybrid turkeys [Converter (Hendrix Genetics BV, NL)] were monitored. Flocks were housed at 13 days of age and a total of 570 birds were included in the four trials (Table 1). The birds were mixed sex. Initially, flocks were raised in a brooder and then transferred to grow-out houses from 8 weeks of age until the end of the production cycle, which occurred at approximately 18 weeks of age. The turkeys were raised on wood shavings and/or rice hulls. The brooder was warmed to 30 °C before the young turkeys arrived and each week the temperature was reduced about 3–4 °C. The birds were fed ad libitum and had fresh water available during the entire experimental period. All houses had mesh windows on the sides of the buildings, and were equipped with automatic drinkers and manual feeders, and with manually controlled ventilation systems. Natural light, which entered the house through the windows, was supplemented with artificial lighting (incandescent lamps) for a total of 23 h of light per day. The brooder house measured 3.0 m × 3.0 m and grow-out houses measured 10.0 m × 15.0 m; all houses in the study had these dimensions.

### 2.3. Data Collection

Flock sounds were recorded in the brooder and the grow-out houses during all trials using a laptop computer coupled to a microphone with a high frequency response (Sennheiser K6/ME4000, frequency response 40–20,000 Hz ± 2.5 db), which was held in place using a short tripod. Sound recordings were collected using the microphone placed at a height between 0.2 m and 0.4 m depending on the height/age of the turkeys.

Flock sounds were recorded for 11 days in each trial for 30 min each day at 13-14 h without human presence in the place. The days when recordings took place are reported in Table 1. On the same days of recordings, the weights of the turkeys also were recorded. For weighing, 30 birds from the mixed sex were randomly selected and were manually weighed using a high-precision (0.005 kg) digital scale (A&D Company, Ltd., Tokyo, Japan).

### 2.4. Sound Analysis

In each trial, a total of 550 sounds from 11 days of recordings (50 sounds per day, 2200 in total), were chosen at random and selected for analysis. Sound recordings were manually analyzed and labelled using Adobe^®^Audition^TM^ CS6 software (Adobe, San Jose, CA, USA, www.adobe.com). The first five minutes of recordings were not included in the sound analysis because the behavior of the turkeys might have been influenced by the setup of the equipment. Every recording file was segmented into shorter files of 5 min to simplify the sound analysis. Sound labelling involved the extraction of animal sounds and general sounds coming from the whole flock based on the amplitude and frequency of the sound signals [2].

**Figure 1:**
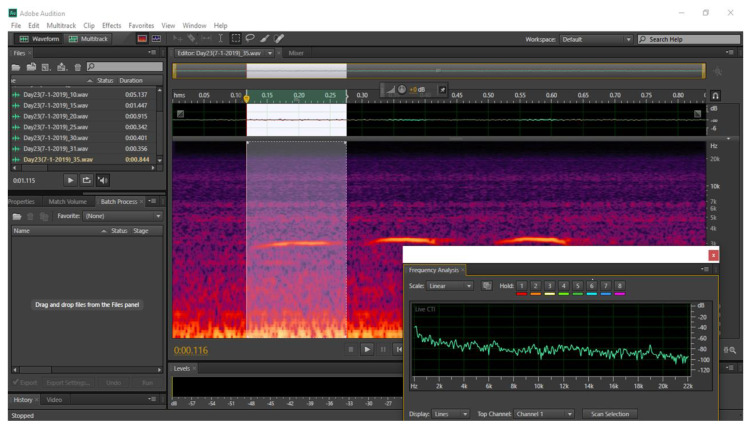
Screenshot of a specific vocalization is highlighted for being analyzed using Adobe^®^ Audition ^TM^ CS6 software (Adobe, San Jose, CA, USA, www.adobe.com). In the window the time-frequency vocalization graph is shown, while the inset represents the frequency analysis.

Labelling is a manual procedure based on acoustic analysis combined with visual spectral analysis. In this study, labelling was used to extract fragments of sounds from each recording. This involved selecting and extrapolating the sounds that were classified as useful vocalization sounds (avoiding those associated with conflict and stress) via audio analysis and visual observation of the spectrogram, following guidance of Ferrari et al. [20]. Using the CS6 software, each sound was identified and analyzed using time (x-axis) and frequency (y-axis). The option “Fast Fourier Transform” (FFT) was used to obtain the PF from the frequency analyses of each sound. A hamming window in the FFT dimension was used as shown Figure 1. The PF represents the frequency of maximum power and was obtained manually using the FFT option. The frequencies lower than 1000 Hz were removed because observations of the spectrograms indicated these sounds were background noise.

### 2.5. Data Processing

#### 2.5.1. Estimation of the Relationships among Age, Bird Weight and PF in Each Trial

For each trial, means and standard error for the weights and PF of the sound emitted by the turkeys at different ages were estimated using the PROC GLM procedure in SAS 9.3 statistical analysis software (SAS, Cary, NC, USA, www.sas.com). The daily means for the weights and corresponding PF at different ages were used to estimate the linear regression and correlation in the four trails, separately. The PROC REG procedure in the SAS 9.3 software was used to analyze data from each trial to obtain regression models that predicted both the weight and PF using the age, and that predicted the weight using the PF. The PROC procedure in the SAS 9.3 software was used to analyze data from each trial and estimate the correlations among PF, age and weight. The regression models to predict the body weight using the PF from each trial were then assessed using the data obtained in the other trials.

For each trial, the observed mean weight and the mean weight predicted by the regression models were obtained. Accuracy of the regression models was evaluated using the coefficient of determination (r^2^) which describes the proportion of the variance in the dependent variable that is predictable from the independent variable, it ranges from 0 to 1, when r = 1 indicates that all the variance of the dependent variable is explained by the independent variable [21], slope of the regression line (b) which if =1 indicates to the model perfectly reproduces the magnitudes of measured data [22], Nash-Sutcliffe efficiency (NSE) that indicates how well the plot of observed versus predicted data fits the 1:1 line, in the case of regression procedures it is ranging between 0 and 1 [22] and when the NSE = 1 corresponds to a perfect match of modelled to the observed data [23], the root mean square error (RMSE) that measures the average prediction error made by the model in predicting the outcome for an observation and the lower the RMSE is in the better the model, and fractional bias (FB) that based on the difference between the predicted and observed values divided by average predicted and observed values and the optimal value of FB is 0, with low-magnitude values indicating accurate model estimation and the positive values indicate model underestimation bias, and negative values indicate model overestimation bias [22].

#### 2.5.2. Estimation of the Relationships among Age, Weight and PF Based on Pooled Data

Analysis of variance (ANOVA) was used to test the differences across variables of age, weight and PF in the four trials. The option SOLUTION with the PROC GLM procedure in SAS 9.3 software was used to estimate the effects of trials on the regression variables estimates.

All the daily means for the bird weights and PF of the sounds in the four trials were used to determine three linear regressions that predicted the weight using age, predicted PF using age, and predicted weight using the PF. To evaluate the differences among trials, the regressions obtained from the pooled data were used to predict the bird weights by the PF in each trial, individually.

The PROC REG procedure in the SAS 9.3 software was used to estimate the linear regression and correlation as explained above. These regression models were then used to predict the weight and PF in all the trials. Accuracy of the regression models was evaluated by the statistics mentioned above and according to Moriasi et al. [22].

#### 2.5.3. Definition and Validation of a Model to Predict Turkeys’ Weight Using the PF of Their Vocalizations

The data obtained in all trials, pooled together, were used to define a prediction model to estimate the weight of the turkeys during their growth based on the PF of their vocalizations. To define and validate the model, the data were processed using Unscrambler 9.7 software (CAMO, Oslo, Norway). All daily means for the weight, PF and age were used in the software’s Cross-Validation procedure to estimate the correlation and regression between weight and PF.

Accuracy of the regression model was evaluated by the statistics mentioned above and according to Moriasi et al. [22].

## 3. Results

### 3.1. Estimated Relationships among Age, Bird Weight and PF in Each Trial

**Figure 2:**
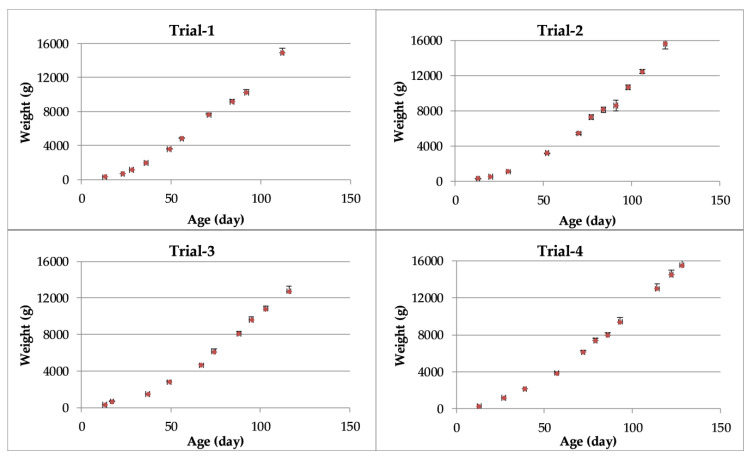
Means and standard error (bars) of turkey weights as a function of age.

Means of the observed turkey weights (g) during the four trials are presented in Figure 2. As expected, body weights increased with increasing bird age in all trials.

**Figure 3:**
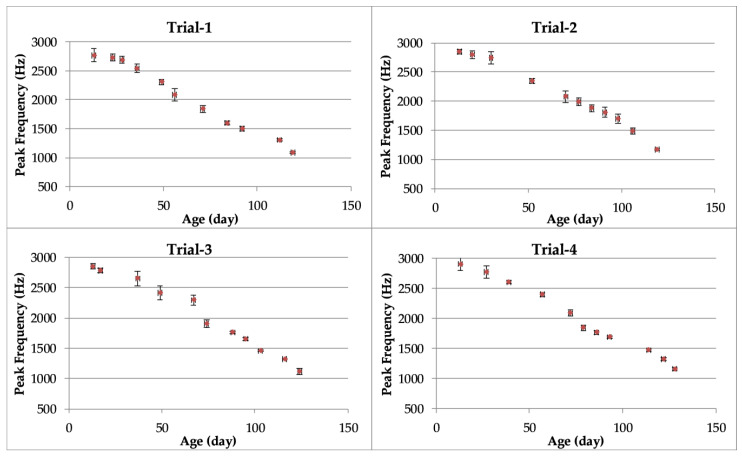
Means and standard error (bars) of the peak frequency of bird vocalizations as a function of turkey age.

The means of the PFs of vocalizations by turkeys (Figure 3) decreased with increasing bird age. The PF values ranged from 2907 to 1084 in the growing periods (days 13 to 128) of all trials.

**Figure 4:**
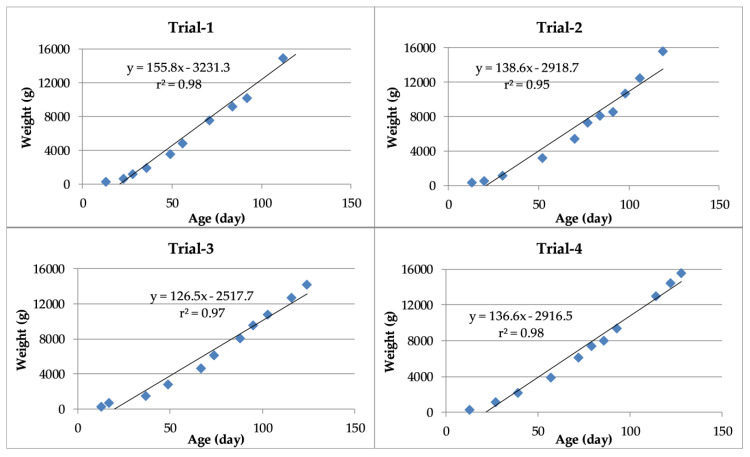
Linear regressions and coefficients of correlation between weight and age of turkeys.

The linear regressions between weight and age of turkeys during the four trials are presented in Figure 4. The constants in the four models ranged from 2517.7 to 3231.3. Furthermore, the coefficients of determination between weight and age were positive and exceeded 95% in each trial.

**Figure 5:**
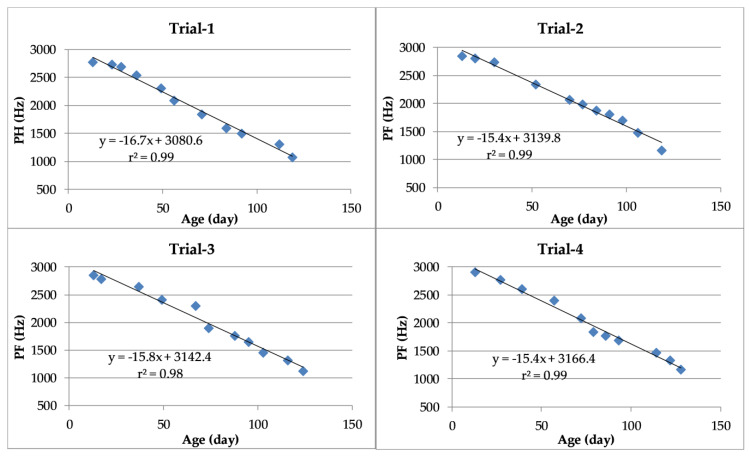
Linear regressions and coefficients of determination between peak frequency (PF) of turkey vocalizations and turkey age.

The linear regressions between the PF of vocalizations and the age of turkeys are shown in Figure 5. The coefficients of determination exceeded 97% in each trial.

**Figure 6:**
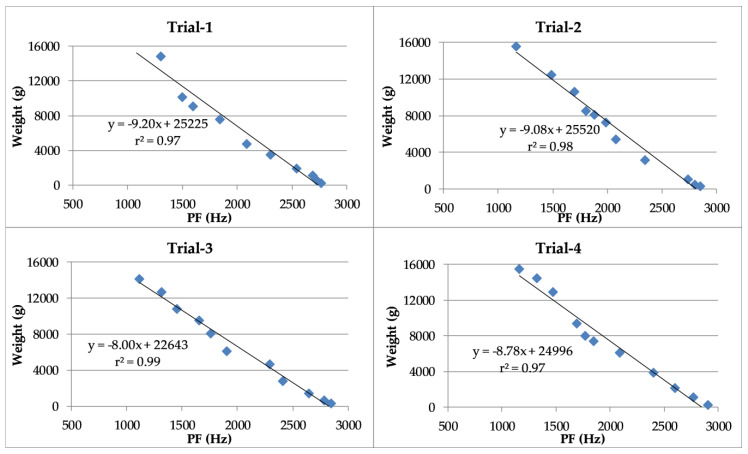
Linear regressions and coefficients of determination between turkey weight and peak frequency (PF) of the vocalizations.

The linear regressions between the weight of turkeys and the PF of their vocalizations are presented in Figure 6. The coefficients of determination exceeded 96% in each trial.

Each regression model shown in Figure 6 was used to predict the weight by PF of turkey vocalizations in the other three trials. The statistics for evaluating the performance of the regression models are presented in Table 2.

### 3.2. Estimated Relationships among Age, Weight and PF Based on Pooled Data

The results of ANOVA to test for significant differences in variables among the four trials showed no statistically significant effects of trials on any variable; thus, the null hypothesis (Ho: T_1_ = T_2_ = T_3_ = T_4_) was accepted and confirmed that the PF of turkey vocalizations was not significantly (*p* < 0.05) different in the four trials.

**Figure 7:**
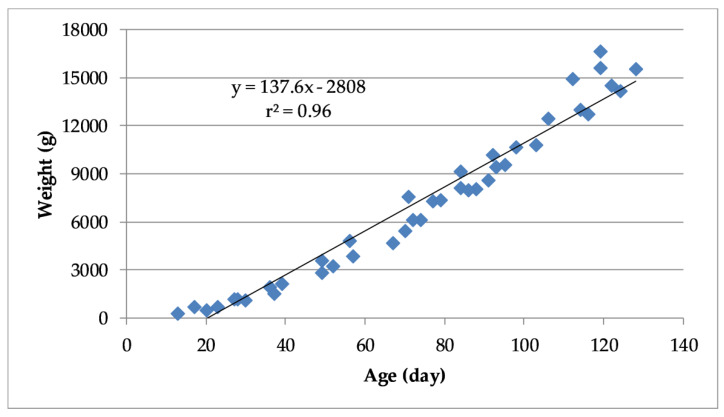
Linear regression and coefficient of determination between turkey weight and age based on pooled data from all trials.

For the pooled dataset, there was a very highly significant positive correlation (r^2^ = 0.96, *p* < 0.0001) between turkey weight and age (Figure 7). The linear regression model developed from the pooled data is described by Equation (1), in which Weight _predicted_ is the projected weight (g) of birds based on their age (Age, days).

Weight predicted = (137.56 × Age) − 2808.1(1)

**Figure 8:**
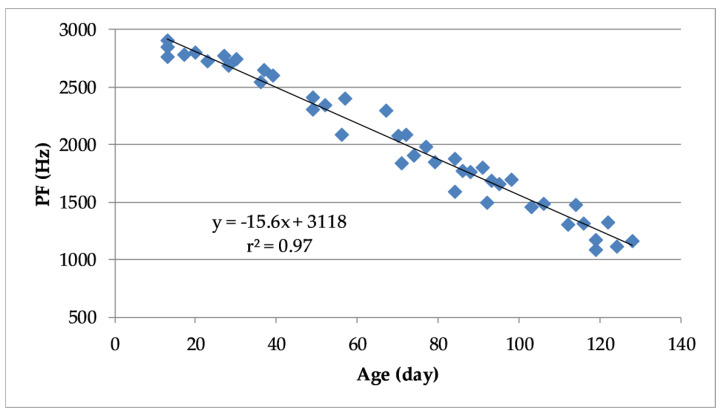
Linear regression and coefficient of determination between peak frequency (PF) of vocalizations and age in turkeys based on pooled data from all the trails.

Similarly, based on the pooled data there was a highly significant negative correlation (r^2^ = 0.97, *p* < 0.001) between the PF of turkey vocalizations and their age (Figure 8). Figure 8 also shows the linear regression model that predicts PF of the vocalization as a function of turkey age.

**Figure 9:**
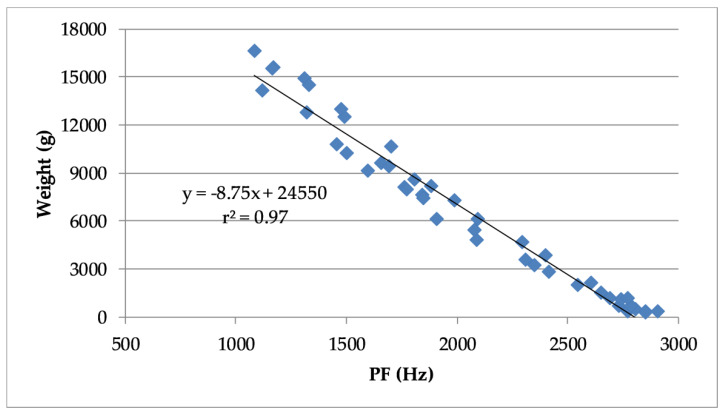
Linear regression and coefficient of determination between turkey weight and the peak frequency (PF) turkey vocalizations based on pooled data from all trails.

Furthermore, using the pooled dataset, it was possible to confirm a significant negative correlation (r^2^ = 0.97, *p* < 0.001) between the PF of the vocalizations and the weight of the turkeys (Figure 9).

The linear regression model for predicting the weight (g) of turkeys using the PF (Hz) of their vocalizations is given as Equation (2):

Weight predicted = (−8.7463 × PF) + 24550(2)

The results of using Equation (2) to predict the weight of turkeys in each trial are presented in Table 3.

Table 3 shows that the coefficient of determination (r^2^), the regression slope (b) and the Nash–Sutcliffe efficiency (NSE) were very close to a value of 1 when applying Equation (2) to data from each trial. These model evaluation statistics suggest that there was a very good match between the predicted weights and the observed weights according to limits suggested by Van Liew et al. [21], Moriasi et al. [22] and Ritter and Muñoz-Carpena [23]. The fractional bias (FB) values were close to 0.0, which also indicated a good match between observed and predicted turkey weights.

### 3.3. Calibration and Validation of a Model to Predict the Weight of Turkeys Using the PF of Their Vocalizations

**Figure 10:**
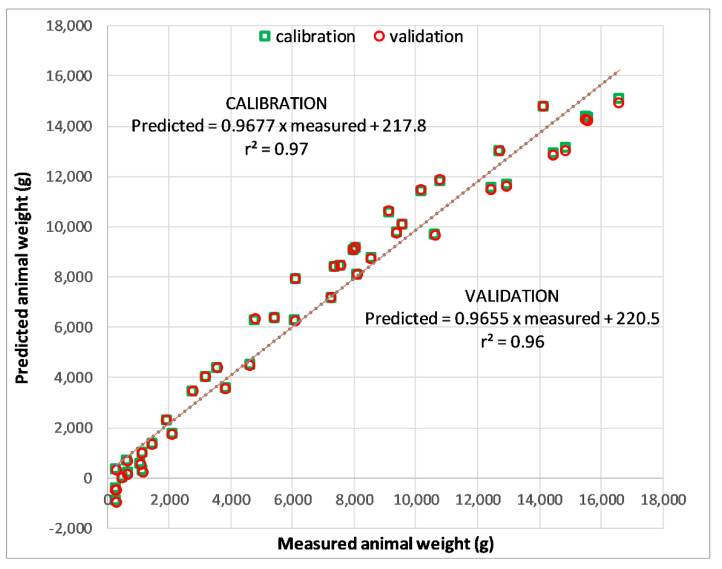
Scatter plot and 1:1 line of perfect agreement between observed turkey weights and weights predicted during calibration and validation.

Scatter plots (Figure 10) show close agreement between observed turkeys weights and weights predicted during calibration and validation.

Most of the weights that were predicted using the PF of the turkey vocalization in all the days through the four trials were very close to the observed weights (Supplementary Files, Table S1).

The model evaluation statistics resulting from the calibration and validation by using cross-validation are reported in Table 4. The assessment produced a high degree of linearity between the predicted and measured turkey weights during both the calibration and validation assessments, which indicated that the model was acceptable [22]. The acceptability of the model was reinforced by values for r^2^ (very close to 1) and FB (close to 0). Although the bias of the model was higher during validation than during calibration (Table 4), this was a typical outcome; nevertheless, the bias was low in both assessments. The RMSE values during calibration and validation were low and differed by only 5.1%, providing further evidence that the model was acceptable [24]. Based on these results, the model was deemed to be an acceptable model through which the PF of turkey vocalizations could be used to predict the weight of turkeys.

## 4. Discussion

### 4.1. Weight-Age Relationship

During the four trials, body weights of turkeys increased as the birds increased in age and were similar to those observed by Yilmaz et al. [4] for the commercial hybrid used in these experiments. Yilmaz et al. [4] reported that the mean body weights of turkeys were 6.500 and 11.172 kg at days 75 and 105 of age, respectively, whereas in the present study, they were 6.551 and 11.314 kg at days 75 and 106, respectively.

There has been increased interest in analyzing the weight-age relationship of animals raised commercially [25]. The changes in body weight as a function of age define the “growth curve” [26]. Equation (1) describes the model developed in this study and is applicable for predicting the weight of turkeys using their age throughout the most growth period (approximately 120 days). The estimation of growth pattern parameters can be important for the economic viability of production. In livestock-breeding, growth patterns are used to determine the general state of health of the animals, to investigate the effects of selection on growth curve parameters, to identify the optimum slaughtering age, to identify the breeding age, and to identify the age of sexual maturity [8]. Equation (1) may be a useful way to obtain indicative bird weights as a function of age in turkeys. Of course, this equation is an empirical equation and it could be applied only for this hybrid turkey and the conditions which this study was conducted.

### 4.2. Weight-Sound Relationship

PF is a very common parameter used in analyses of acoustic variation. The sound analysis in this study was based on the PF that was measured by audio spectra using a FFT. This mathematical function converts a signal to the frequency domain and the output consists of values specifying the amplitudes associated with a sequence of frequency components within an entire signal (called the “power spectrum” or the “magnitude spectrum” of the waveform) [27]. The spectrograms are excellent tools for extracting acoustic features that help visualize and describe the acoustic signals, as well as for comparing sound recordings through frequency contours and identifying sounds of interest [20].

The mean PFs for turkey vocalizations ranged from 2907 Hz (on day 13) to 1084 Hz (on day 128) during the growing period. Analysis of sounds by FFT in a study by Liu et al. [16] revealed that the main frequency range of turkey vocal sounds was between 2500 Hz (at 8 weeks) and 600 Hz (at 16 weeks) during the growing period, while non-vocal sounds ranged in frequency between 10 Hz and 1500 Hz. Thus, there was some overlap of the two types of sounds in the frequency range from 600 to 1500 Hz. As shown in Figure 3, the frequencies of turkey sounds in this study were near 2500 Hz on the days within the first 8 weeks of poult growth, in agreement with values measured by Liu et al. [16].

There was a clear decrease in the PF of turkey vocalizations with increasing age and weight of turkeys in the four trials (and in the resulting pooled dataset). The evaluation statistics for the accuracy of the regression model that was used to predict turkey weight on the basis of PF. Equation (2) showed a highly significant negative correlation between weight and PF. These results are in agreement with results reported for turkeys by Liu et al. [16] and reinforce findings of previous studies that reported changes in animal vocalizations as the animals grow [28]. The PF of broiler chicken vocalizations also is very highly correlated ((*p* < 0.0001)) with the age and weight of birds [17], permitting the use of PF to predict changes in bird weight. All these conform that the modern acoustic and digital signal processing techniques offer solutions to problems and these are widely uses in animal management field [29,30].

Cross-validation, sometimes called “rotation estimation” or “out-of-sample testing”, used to test ability to make predictions of independent observations, showed that the model was acceptable.

It is now generally agreed that vocalizations consist of travelling waves generated by airflow-induced oscillation of elements in the wall of the syrinx that convert some of the airflow’s kinetic energy into acoustic energy [31]. These oscillations are presumably sustained by interaction between the vocal tract and air from respiratory system [32]. Little is known about how the respiratory tract or vocal tract develops to achieve adulthood sound. However, young birds must undergo many changes on their way to adulthood. From a physiological view, young birds are able to coordinate respiratory system and muscular system control of the vocal organ with the fine control of airflow [33]. These developments may explain the causes for the changes shown in the PF of vocalizations as a function of age or body weight. The system for sound production as a function of a bird’s age and growth stage presents an interesting model for exploring the development and control mechanisms in sound generation.

## 5. Conclusions

Even though they are empirical, the results from this study clearly demonstrate that the PF of turkey vocalizations can be used to predict bird weights. This technique will allow a farmer to automatically monitor the growth of turkeys and avoid manual handling, which is difficult and dangerous, both for the birds and the handlers. Although the specific predictive model developed in this study (i.e., Equation (2)) is strictly applicable only to the conditions encompassed in this study, it shows that audio monitoring can serve as means by which to obtain useful indicators about the growth of turkeys. As such, it is a promising technique for the development of an automated growth-monitoring assessment tool for use by turkey farmers. However, further research is necessary to improve the algorithms for predicting bird weight based on the PF of their vocalizations. In the least, these studies should include a variety of study conditions to generalize the applicability of the predictive equations.

## Figures and Tables

**Table 1:** Total number of birds monitored, day numbers (age of turkeys) for making recordings and weighing birds, and the duration of each trial.

Trial No.	Total No. of Birds	Day Numbers for Sound Recording and Bird Weighing	Duration, Days
1	250	13, 23, 28, 36, 49, 56, 71, 84, 92, 112 and 119	13–119
2	120	13, 20, 30, 52, 70, 77, 84, 91, 98, 106 and 119	13–119
3	100	13, 17, 37, 49, 67, 74, 88, 95, 103, 116 and 124	13–124
4	100	13, 27, 39, 57, 72, 79, 86, 93, 114, 122 and 128	13–128

**Table 2:** Estimation the turkey weight using the peak frequency of vocalizations in each trial based on the regression models.

Estimated Weight (G) Using Peak Frequency with the Four Regression Models			Evaluation Statistics				
Models	Mean Observed	Mean Predicted					
Model	Trial-1		r^2^	b	NSE	RMSE	FB
Trial-2	6517.8	6196.7	0.962	1.022	0.975	757.96	−0.97
Trial-3	6318.6	6597.0	0.933	1.272	0.866	1604.23	−1.77
Trial-4	7553.2	7925.2	0.984	1.190	0.943	928.60	−0.06
Model	Trial-2						
Trial-1	6534.5	6842.4	0.970	0.933	0.970	1010.391	0.085
Trial-3	6318.6	6902.6	0.966	1.221	0.880	1507.981	−1.184
Trial-4	7553.2	7925.2	0.984	1.190	0.943	928.60	−0.06
Model	Trial-3						
Trial-1	6534.5	6815.4	0.943	0.739	0.913	1,626.33	0.75
Trial-2	6517.8	6558.5	0.962	0.778	0.939	1,182.72	0.82
Trial-4	7553.2	7874.4	0.984	0.906	0.979	584.48	0.13
Model	Trial-4						
Trial-1	6534.5	6393.8	0.943	0.809	0.967	1312.14	0.23
Trial-2	5609.6	5405.7	0.962	0.851	0.978	994.80	−0.05
Trial-3	6318.6	6446.1	0.933	1.060	0.981	913.08	−0.71

r^2^ = coefficient of determination, b = slope of regression line, NSE = Nash-Sutcliffe efficiency, RMSE = the root mean square error, FB = fractional bias.

**Table 3:** Estimated turkey weights in each trial and evaluation statistics of the accuracy for using Equation (2).

Estimated Weight (G) Using Peak Frequency (Equation (2))			Evaluation Statistics				
Trial no.	Mean Observed	Mean Predicted	r^2^	b	NSE	RMSE	FB
Trial-1	6445.8	6692.6	0.966	0.918	0.961	1,073.978	0.079
Trial-2	6656.4	6376.5	0.982	0.945	0.977	739.867	−0.322
Trial-3	6488.7	6891.1	0.986	1.078	0.970	810.424	−0.208
Trial-4	7393.7	7022.8	0.970	0.965	0.964	959.408	−0.435

r^2^ = coefficient of determination, b = slope of regression line, NSE = Nash-Sutcliffe efficiency, RMSE = the root mean square error, FB = fractional Bias.

**Table 4:** Model evaluation statistics during calibration and cross-validation when predicting the weight of turkeys using the PF of their vocalizations.

Evaluation Statistics	Calibration	Validation
r^2^	0.97	0.96
Slope	0.967715	0.965523
NSE	0.968	0.964
RMSE	905.27	951.36
FB	0.002	0.179

r^2^ = coefficient of determination, b = slope of regression line, NSE = Nash-Sutcliffe efficiency, RMSE = the root mean square error, FB = fractional Bias.

## Supplementary Materials

The following are available online at https://www.mdpi.com/2076-2615/10/5/866/s1, Table S1: Observed and predicted weights in calibration and validation model based on PF.
